# Glomangioma of the Knee: A Case Report

**DOI:** 10.1155/cro/5971036

**Published:** 2026-06-20

**Authors:** Grayson Bastin, Brandon Hartman, Haley Prough, Jason Cochran

**Affiliations:** ^1^ McLaren Greater Lansing Hospital, Lansing, Michigan, USA, mclaren.org; ^2^ Michigan State University, East Lansing, Michigan, USA, msu.edu; ^3^ University of Michigan Health - Sparrow Hospital, Lansing, Michigan, USA; ^4^ Michigan Orthopedic Center, Lansing, Michigan, USA

**Keywords:** anterior knee pain, extradigital glomus tumor, glomangioma, soft tissue tumor, surgical excision

## Abstract

Glomus tumors are rare, benign mesenchymal neoplasms arising from the glomus body, typically found in the digits and involved in thermoregulation. We present a 54‐year‐old male with a 5‐year history of sharp, throbbing anterior knee pain worsened by activity. Initial imaging showed only mild osteoarthritis without soft tissue abnormality. Persistent, localized symptoms prompted further evaluation, revealing a small subcutaneous mass anterior to the patellar tendon. Surgical excision yielded a well‐circumscribed ovoid lesion, confirmed as a glomangioma on histology. Following the procedure, the patient experienced complete and sustained symptom resolution. This case highlights the diagnostic challenges of extradigital glomus tumors and underscores the importance of early identification and surgical intervention to reduce patient morbidity.

## 1. Introduction

Glomus tumors are rare, benign mesenchymal neoplasms that arise from the glomus body, which is a specialized neuromyoarterial structure located within the dermis of the digits and involved in thermoregulation [1]. These tumors represent approximately 1.6% of all soft tissue neoplasms in the extremities, with a strong predilection for the subungual region of the hand and a notable female predominance (5:1 female‐to‐male ratio) [2–4]. Extradigital glomus tumors, by contrast, are uncommon and more frequently misdiagnosed, often presenting with a male predominance (1:4 female‐to‐male ratio) [5].

Histologically, glomus tumors are composed of three main components: glomus cells, vasculature, and smooth muscle cells. They are classified into three subtypes, solid glomus tumors (75%), glomangiomas (20%), and glomangiomyomas (5%), based on the relative prominence of these elements [6]. Clinically, patients typically present with a classic triad of intense paroxysmal pain, point tenderness, and cold sensitivity. Although most cases are found subungually, glomus tumors have also been reported in visceral locations such as the lung and gastrointestinal tract, as well as in extradigital sites on the extremities [7–9].

Due to their rarity and often atypical presentation outside the nail bed, extradigital glomus tumors are underrecognized clinically, frequently resulting in delayed diagnosis and treatment [5]. This case highlights an unusual presentation of an extradigital glomus tumor and underscores the importance of clinical suspicion, especially in the context of chronic unexplained pain and point tenderness.

## 2. Case Presentation

A 54‐year‐old male presented to the clinic in October 2020 with a five‐year history of anterior right knee pain. The pain was described as sharp, throbbing, and shooting in nature, worsened by activity, and occasionally accompanied by a popping sensation and episodes of the knee giving way. Notably, there was no history of trauma or identifiable inciting event.

The patient denied any significant medical, social, or family history relevant to his knee pain. On physical examination, there were no skin changes or ulcers over the knee. His right knee demonstrated a full range of motion from 0° to 135°, with 4/5 muscle strength across all muscle groups and intact sensation. Special tests including McMurray, varus/valgus stress, anterior and posterior drawer, patellar compression, and patellar apprehension were negative. Crepitus was appreciated over the patellofemoral joint throughout range of motion, and there was exquisite point‐tenderness along the inferior aspect of the patella and patellar tendon.

A right knee MRI performed 3 years prior showed mild tricompartmental osteoarthritis and nonspecific degenerative changes, with no evidence of acute internal derangement or obvious soft tissue abnormality per the report. Based on clinical and imaging findings, the patient was diagnosed with mild right knee osteoarthritis and associated inferior patellar tendonitis, also involving the tibial tubercle. He received an intraarticular cortisone injection in clinic and was advised to rest, modify activity, apply ice and elevation, and take Tylenol or NSAIDs as needed.

The patient returned to the clinic 28 months later in February 2023 with worsening symptoms. He reported increased swelling and severe anterior knee pain rated 10/10, although his physical examination remained unchanged from his prior visit. A repeat noncontrast MRI of the right knee was obtained. According to the radiology report, the study demonstrated a new small radial tear of the lateral meniscus, mild interval progression of tricompartmental chondromalacia, and an unchanged small Baker′s cyst containing loose bodies. No extracapsular soft tissue abnormalities were noted in the radiologist′s interpretation. However, on independent review by the treating physician, a small subcutaneous soft tissue density was identified anterior to the patellar tendon and proximal to the tibial tubercle. The lesion appeared hypointense on T1 and hyperintense on T2 sequences, raising concern for a mass versus fluid collection in the region corresponding to persistent point tenderness on examination (Figure 1). To further characterize the finding, an ultrasound of the right knee was performed, which confirmed the presence of a solid, subcutaneous hypoechoic mass inferior to the patella, measuring 1.0 × 0.6 × 0.3 cm (Figure 2). The appearance and location were concerning for possible subcutaneous nerve involvement, raising suspicion for a nerve sheath tumor or neuroma.

**Figure 1:**
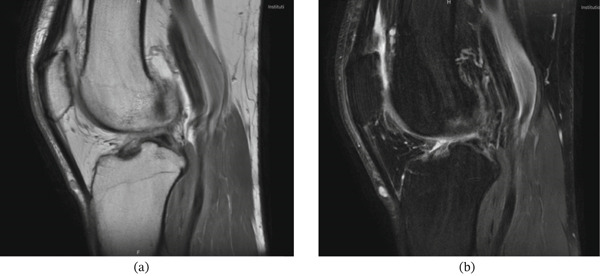
(a) Figure details T1‐weighted sagittal MRI of the right knee demonstrating a small subcutaneous mass anterior to the distal insertion of the patellar tendon, appearing hypointense on T1 imaging. (b) Figure details T2‐weighted sagittal MRI of the right knee demonstrating the same lesion, appearing hyperintense on T2 imaging.

**Figure 2:**
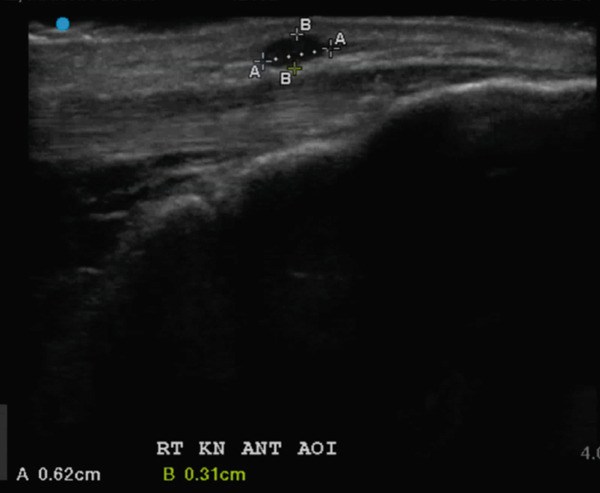
Right knee ultrasound demonstrating a solid, subcutaneous hypoechoic mass inferior to the patella, measuring 1.0 × 0.6 × 0.3 cm.

The patient underwent surgical excision of the mass in May 2023. Intraoperatively, a well‐circumscribed ovoid nodule measuring 2.1 × 1.5 × 0.7 cm was excised from the soft tissue inferior to the patella (Figure 3). Histopathological analysis revealed glomus cells positive for smooth muscle actin (SMA) and vimentin, confirming the diagnosis of a glomangioma (Figure 4).

**Figure 3:**
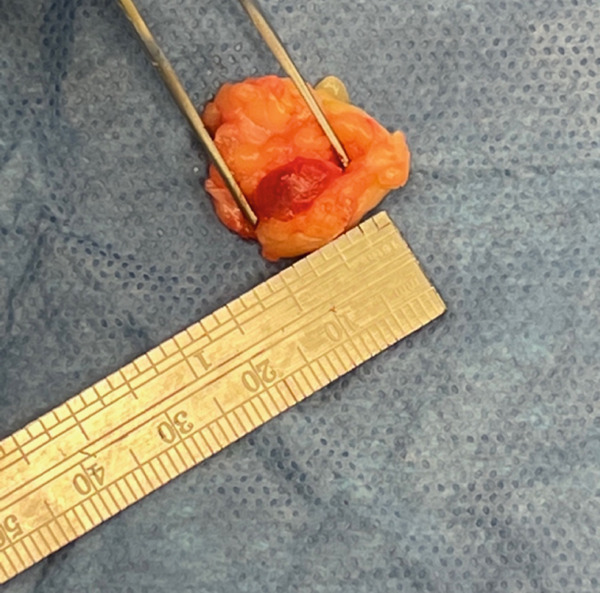
Mass pictured after surgical excision. Tan, ovoid nodule within yellow, lobular soft tissue measuring 0.7 × 0.5 × 0.3 cm.

**Figure 4:**
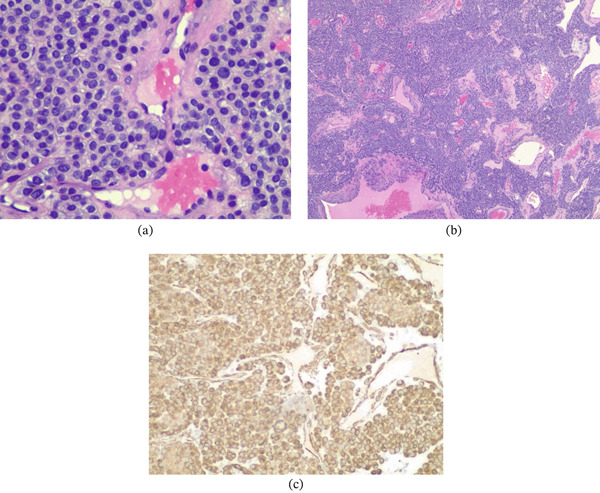
(a) High‐power H&E stain demonstrates glomus cell aggregates surrounding small blood vessels. (b) Low‐power H&E stain demonstrates glomus cell aggregates surrounding small blood vessels. (c) High‐power SMA stain demonstrates glomus cells immunoreactive to smooth muscle actin.

Following excision, the patient reported sustained relief of his anterior knee pain, marking the resolution his long‐standing clinical complaint.

## 3. Discussion

Glomus tumors were first described in 1812 by Wood [4] as painful subcutaneous nodules. Although typically subungual in origin, extradigital glomus tumors can be difficult to diagnose due to their low incidence, atypical locations, and nonspecific symptoms [5, 8, 10]. Prior to 2020, only 91 extradigital glomus tumors had been reported, and among this minority of glomus tumors knee involvement was the most common (41.3%), followed by the hand, shoulder, and vertebral column [9].

Glomus tumors typically present with the classic triad of cold hypersensitivity, paroxysmal pain, and point tenderness. However, extradigital forms can lack these hallmark features [10, 11]. These lesions often present grossly with a reddish‐purple hue in patients with and without pain symptoms [12]. Patients with knee involvement often present with vague anterior knee pain, sometimes for years, leading to misdiagnoses such as osteoarthritis, tendinopathy, or meniscal injury. Wang, et al. [9] performed a review of published literature, which demonstrated an average delay of approximately 3 years between symptom onset and diagnosis of extradigital glomus tumor.

Reported sites of glomus tumors within the knee include the medial and lateral joint lines, the quadriceps and vastus lateralis muscles, overlying Baker′s cysts, the anterior knee, and within the patellar ligament [7, 9, 10]. A recent case has also described a glomus tumor in the same location as in our report—superficial to the patellar tendon at its distal insertion [13]. This variability in location, combined with subtle clinical features, may complicate the diagnostic process.

Due to the rarity and ambiguous presentation, diagnosis requires a high index of suspicion. MRI is considered the most sensitive imaging modality, that typically shows a small, well‐circumscribed, T1‐hypointense, and T2‐hyperintense lesion with postcontrast enhancement. Ultrasound can be a useful adjunct in identifying superficial lesions and assessing vascularity, but is operator‐dependent and has been shown to be less sensitive than MRI [10, 12]. Histopathologic confirmation remains the diagnostic gold standard [11].

The treatment of choice for glomus tumors, including those around the knee, is complete surgical excision, which typically results in immediate pain relief and resolution of symptoms. Recurrence rates after surgical resection are low, ranging from 5% to 10% in the literature [7, 10]. Malignant forms of glomus tumors are considered extremely rare and are generally not a concern, with as low as 1% of glomus tumors reported as malignant [14].

## 4. Conclusion

Extradigital glomus tumors, though rare, should be included in the differential diagnosis for patients presenting with localized knee pain, that is unresponsive to conventional treatments for more common conditions such as osteoarthritis or tendinopathy. Due to their atypical presentation and low incidence, these tumors are frequently misdiagnosed, often resulting in prolonged symptoms and frustration for both patients and healthcare providers. Early clinical recognition and appropriate imaging are essential to avoid delays in diagnosis and to facilitate definitive surgical management, which typically results in complete symptom resolution.

## Author Contributions

G.B.: data curation, investigation, literature review, writing of the original draft. H.P.: data curation, investigation, literature review. B.H.: literature review, writing of the original draft. J.M.C.: conceptualization, supervision, clinical interpretation, project administration, critical review and editing of the manuscript.

## Funding

No funding was received for this manuscript.

## Disclosure

All authors have revised the manuscript and approved the final version for publication. No artificial intelligence was used in the preparation, drafting, analysis, or revision of this case report. The manuscript represents the original work of the authors.

## Consent

Written informed consent was provided by the patient for the publication of this case report and any accompanying clinical data and images.

## Conflicts of Interest

The authors declare no conflicts of interest.

## Data Availability

The data that support the findings of this study are available from the corresponding author upon reasonable request.
